# Robust and Reproducible Quantification of the Extent of Chest Radiographic Abnormalities (And It’s Free!)

**DOI:** 10.1371/journal.pone.0128044

**Published:** 2015-05-21

**Authors:** Ana Requena-Méndez, Edelweiss Aldasoro, Jose Muñoz, David A. J. Moore

**Affiliations:** 1 ISGlobal, Barcelona Ctr. Int. Health Res. (CRESIB), Hospital Clínic—Universitat de Barcelona, Barcelona, Spain; 2 TB Centre, London School of Hygiene and Tropical Medicine, London, United Kingdom; Wexner Medical Center at The Ohio State University, UNITED STATES

## Abstract

**Rationale:**

Objective, reproducible quantification of the extent of abnormalities seen on a chest radiograph would improve the user-friendliness of a previously proposed severity scoring system for pulmonary tuberculosis and could be helpful in monitoring response to therapy, including in clinical trials.

**Methods:**

In this study we report the development and evaluation of a simple tool using free image editing software (GIMP) to accurately and reproducibly quantify the area of affected lung on the chest radiograph of tuberculosis patients. As part of a pharmacokinetic study in Lima, Peru, a chest radiograph was performed on patients with pulmonary tuberculosis and this was subsequently photographed using a digital camera. The GIMP software was used by two independent and trained readers to estimate the extent of affected lung (expressed as a percentage of total lung area) in each radiograph and the resulting radiographic SCORE.

**Results:**

56 chest radiographs were included in the reading analysis. The Intraclass correlation coefficient (ICC) between the 2 observers was 0.977 (p<0.001) for the area of lung affected and was 0.955 (p<0.001) for the final score; and the kappa coefficient of Interobserver agreement for both the area of lung affected and the score were 0.9 (p<0.001) and 0.86 (p<0.001) respectively.

**Conclusions:**

This high level of between-observer agreement suggests that this freely available software could constitute a simple and useful tool for robust evaluation of individual and serial chest radiographs.

## Introduction

Chest radiographs (CXR) provide valuable information regarding extent and progression in many respiratory diseases. Accordingly, for the study of specific illnesses such as occupational lung diseases the utility of chest radiology has been greatly improved by the application of standardized reading methodology[1]. Different methodologies, such as the Chest Radiographic Reading and Reporting System[2], have been proposed to standardize CXR reading for TB and other lung diseases, and also for grading the severity of CXR abnormalities[3].

Chest radiography is a rapid examination suitable for on-site interpretation with a high sensitivity when any abnormality is considered[4]. However, the heterogeneous CXR manifestations of pulmonary TB can lead to inconsistencies in CXR interpretation. Similarly CXR reading is somewhat subjective, so CXR interpretation is highly reader-dependent which can contribute to inter- and intra-observer differences[5,6] and is also dependent upon the expertise of the reader[7]. There have been several attempts to automate reading of CXR by computers[4,8] although it is challenging, particularly due to the low specificity[4].

Recently a simple method for grading chest radiography (CXR) severity in adults diagnosed with sputum smear positive pulmonary tuberculosis (TB) was designed and validated, and shown to correlate with baseline and clinical and microbiological severity and response to treatment[9].

This is likely to be of particular relevance for the evaluation of CXR in clinical trials, where precise, accurate and reproducible data is particularly important. A simple equation was generated to develop the CXR score as follows: proportion of total lung affected (%) + 40 if cavitation is present. This score was able to predict 2-month sputum smear status. To grade the percentage of affected lung, visual estimation of the extent of opacification, cavitation or other pathologies as a percentage of visible lung fields is made.

However, as Ralph *et al* acknowledge, a significant limitation of this method is the low rate of inter-observer agreement in CXR assessment which was low overall, although more substantial agreement was achieved for some variables after adjusting kappa values for variable prevalence and reporter bias[9]. The concordance among the total amount of lung affected was 0.85 (95% limits of agreement 28.2% -22.46%).

Moreover, the poor agreement between radiologists and clinicians has been also reported elsewhere[5,6].

This difficulty (in reproducibly estimating extent of radiographic abnormaliy) can be overcome using novel radiologic software which is capable of accurately measuring a determined area of a radiological digitalized image, giving a precise percentage of lung affected instead of a visual estimation. However this software is not usually available in the field and CXRs are often not performed in a suitable digital X-ray system. Using a standard digital camera, a digital picture of a conventional CXR may be obtained although this file is usually not compatible with digital X-ray software. We have developed a simple methodology based on free image editing software (GIMP, http://www.gimp.org/), which can read any type of digitalized image and provides simple capability to measure selected areas of an image. The objective of this sub-study was to evaluate the reproducibility of lung area estimation using this tool in tuberculosis patients.

## Methods

### Study methods

**Fig 1:**
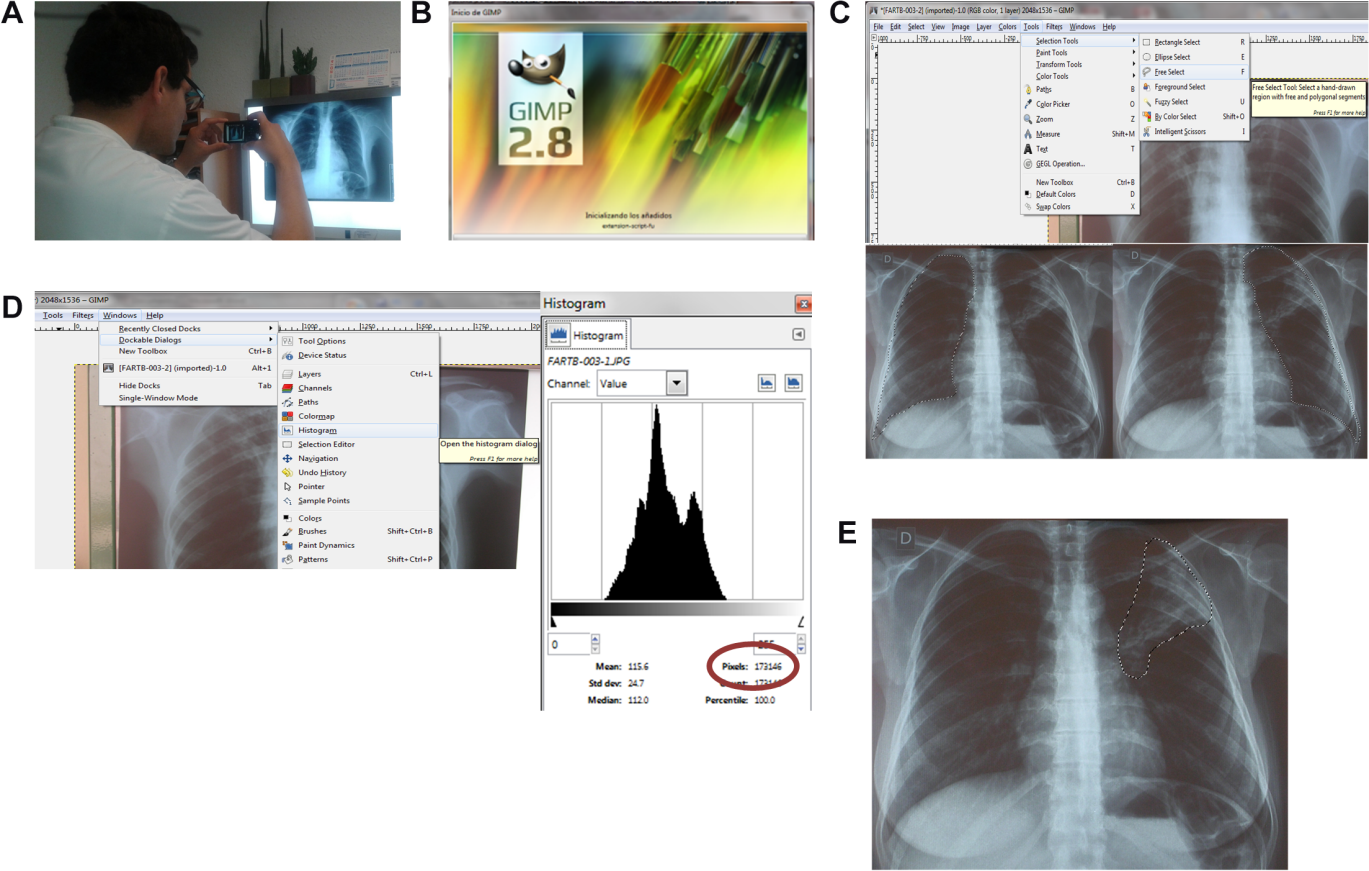
Flowchart of the followed methodology. A. Health worker taking a photo of a radiograph. B. Opening JPEG files with GIMP software. C. Selection of the area. D. Pixel quantification. E. Selected “affected area.”

In a study of TB drug pharmacokinetics patients diagnosed with and treated for pulmonary TB in south Lima, under the DOTS programme of the Peruvian National TB programme, were invited to participate from July to December of 2009. As part of this study, a CXR was performed to all patients to assess cavitation and extent of the disease. All CXR films were digitalized into JPEG files by taking a photograph with conventional digital camera (See Fig 1). The digital image capture was performed by the same person, with the same camera and in the same place for all the CXRs. All CXR films were the same size and the distance from the digital camera to the films was established when the LCD monitor or the viewfinder of the camera framed the whole image. The zoom was not used in order to retain the maximum resolution of the image.

CXRs were coded and stored in a computer at the laboratory offices of Universidad Peruana Cayetano Heredia (UPCH).

### Ethics statement

The study protocol and the consent form were approved by the ethics committee of UPCH and Dirección de Salud-II (DISA II) Lima Sur (regional Ministry of Health). All patients gave written informed consent to participate in the study. The individual from the picture (Fig 1A) in this manuscript has given written informed consent (as outlined in PLOS consent form) to publish these case details.

### CXR evaluation

Two independent raters, blinded to the other’s scores, evaluated the CXRs of study participants. Both were physicians specialized in Internal Medicine with more than 8 years of experience in clinical practice. For the CXR reading, they opened the JPEG files of each radiograph using the free software GNU Image Manipulation programme (GIMP 2.8). The GIMP software is available, with installation instructions, at: http://www.gimp.org/downloads/

Before commencing data collection, the two researchers involved in the study received brief training of 30 minutes about the use of the GIMP software, specifically about how to use the different commands of the software. This software permits determination of a selected area of an image by measuring the number of pixels enclosed in the selected area. The procedure is as follows:

1. > Free selection tool command (a command that permits to select a determined area), (see Fig 1B) then use the mouse to draw a polygon around area of interest.
2. > Dockable Dialogs > histogram (the command that permits to measure this area in pixels) (see Fig 1C), a determined number of pixels is obtained.
3. Enter data into a simple excel spreadsheet with built in equations that automatically calculate percentage of lung area affected and the score.

Accordingly, the pixels of a selected “affected-lung “area (See Fig 1D) can be compared with the pixels o the total area of lungs (this would be the 100%) in the radiography (see Fig 1B) and the percentage of the lung affected can be calculated using a simple rule of three.

This methodology was applied to evaluate the lung affected area in each radiograph and derive a number representing the percentage of lung affected. Readers judged whether cavitation was visualized and added 40 if this was the case, to determine the final score for each radiograph, according to the method developed by Ralph et al,[9]. A data-base model for data-entry can be found in Tables A and B in S1 File.

### Statistical analysis

The agreement between the raters was calculated using an intraclass correlation coefficient (ICC) with a two-way mixed model and with 95% confident interval. ICC was interpreted as poor (0–0.5), moderate (0.5–0.75), good (0.75–0.9) and excellent (0.91–1) according to Portney [10]. Moreover, the variable “Proportion of lung affected” was categorized into 4 different levels of lung affectation: <25%, 25–50%, >50–75% and >75%. The “score” variable was also categorized into 4 different levels: <12.5, 12.5–25, >25–50 and >50. In both cases, the interobserver agreement (IOA) beyond chance was evaluated by calculation of kappa coefficient. Data were analysed using STATA ver. 12.

## Results

**Fig 2:**
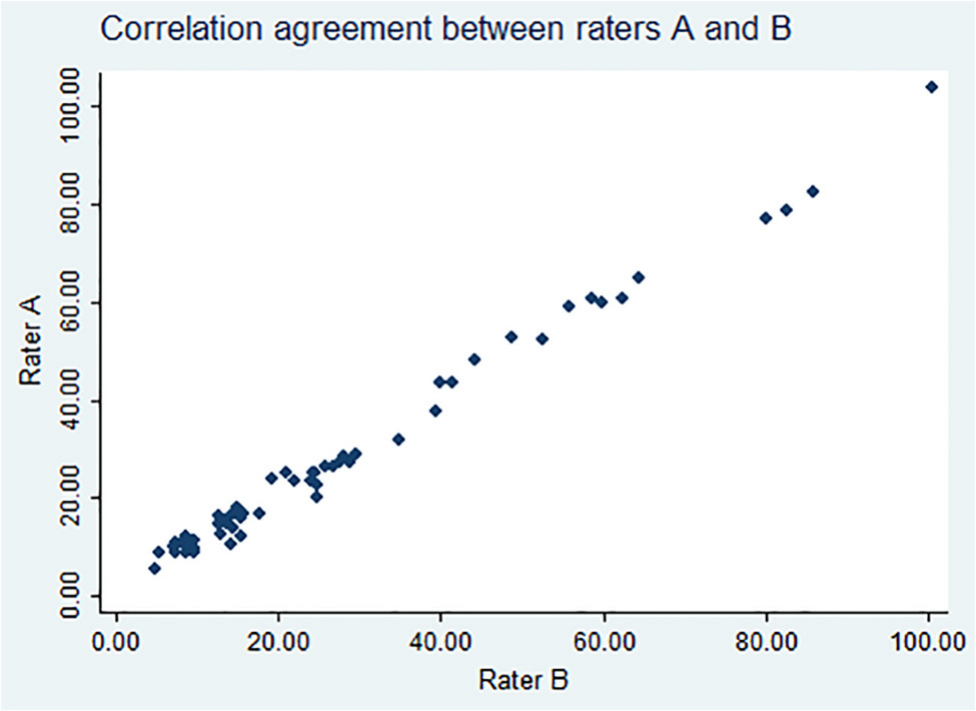
Intraclass correlation agreement between rater A and B.

60 participants were included in the TB – pharmacokinetic study although the CXR was only performed in 56 which were therefore included for the purpose of this study. The raw data can be found in Table C in S1 File. ICC between the 2 observers was 0.923 (0.872–0.954, p<0.001) for the determination of the total area of the lungs (pixels) of each CXR and 0.977 (0.961–0.986, p<0.001) for the area of the lung affected. ICC was 1 when the presence of cavitation was evaluated. When the final composite score was determined, the ICC between the 2 raters was 0.995 (0.991–0.997, p<0.001) (Fig 2). Kappa coefficient for IOA of the score was 0.86 (p<0.001) and when the proportion of lung affected was evaluated, kappa coefficient was 0.9 (p<0.001).

## Discussion

An objective, reproducible and standardized interpretation of chest radiographs for the detection of active pulmonary tuberculosis is crucial in the final evaluation of the severity of the disease and assessment of therapeutic response and the need for a universal and standard system for CXR reporting in TB is acknowledged[9]. The score developed by Ralph *et al*. is indeed a simple tool that can be used where a numerical score is required for the purpose of comparing radiographic severity between adults with smear-positive pulmonary TB and also to monitor an individual’s improvement over time (e.g. to assess drug efficacy in clinical trials). However, calculation of this score requires an assessment of the proportion of lung affected which is subject to significant inter-observer variability [6]. A more robust and reproducible way to define this metric would be very helpful. In health settings where digital systems to perform CXR have been implemented this drawback is substantially reduced since novel software specific for CXR reading permits selection and measurement of polygons or areas. However, such facilities are frequently not available in the majority of resource-constrained countries with the highest burden of TB.

We propose that this alternative tool in which the hard copy chest radiograph film can be captured with a simple digital camera and then read by free software to measure affected areas of the image provides a useful tool for objective, reproducible assessment. Any image processing software could then be used. The high inter-observer agreement of 2 different raters, clinicians but not experienced radiologists, demonstrates the applicability of this tool in the objective interpretation of chest radiographs in a setting representative of clinical practice.

We acknowledge that the lack of an external reference standard with which to compare the observers’ ratings may be regarded as an inherent limitation of our study design. However, demonstrating the reliability and agreement does not require such an external ‘gold standard’ as comparisons are done between and within observers, rather than with an external reference standard, as in diagnostic accuracy studies[11].

## Conclusions

Our findings demonstrate excellent inter-observer agreement in the interpretation of the extent of chest radiographic abnormality in smear-positive pulmonary TB patients. The use of the free and simple-to-use GIMP software should be considered when it is desirable or necessary to quantify the affected proportion of the lung (acknowledging the two-dimensional nature of a CXR). Similarly it may be helpful for both single use or serial review of CXR severity scoring in adults with smear-positive pulmonary TB.

## Supporting Information

S1 File Table A: Instructions for completion of the spreadsheet to calculate proportion of lung affected. Table B: Database model. Table C: Database of the study.
(XLSX)
